# THE LARGEST WESTERN EXPERIENCE WITH HEPATOPANCREATODUODENECTOMY: LESSONS LEARNED WITH 35 CASES

**DOI:** 10.1590/0102-6720201600010005

**Published:** 2016

**Authors:** Eduardo de Souza Martins FERNANDES, Felipe Tavares de MELLO, Joaquim RIBEIRO-FILHO, Asterio Pinto do MONTE-FILHO, Moacir Martins FERNANDES, Romulo Juventino COELHO, Monique Couto MATOS, Antonio Augusto Peixoto de SOUZA, Orlando Jorge Martins TORRES

**Affiliations:** 1 Department of Surgery, Federal University of Rio de Janeiro, Rio de Janeiro, RJ; 2 Department of Surgery and Transplantation of Rio de Janeiro, Adventist Hospital, Rio de Janeiro, RJ; 3 Department of Digestive Surgery, Pio XII Hospital, São Jose dos Campos, SP; 4 Department of Surgery, Federal University of Maranhão, MA, Brazil

**Keywords:** Pancreatic Neoplasms, Hepatectomy, Pancreatectomy

## Abstract

**Background:**

Hepatopancreatoduodenectomy is one of the most complex abdominal operations mainly indicated in advanced biliary carcinoma.

**Aim:**

To present 10-year experience performing this operation in advanced malignant tumors.

**Methods:**

This is a retrospective descriptive study. From 2004 to 2014, 35 hepatopancreatoduodenectomies were performed in three different institutions. The most common indication was advanced biliary carcinoma in 24 patients (68.5%).

**Results:**

Eighteen patients had gallbladder cancer, eight Klatskin tumors, five neuroendocrine tumors with liver metastasis, one colorectal metastasis invading the pancreatic head, one intraductal papillary mucinous neoplasm with liver metastasis, one gastric cancer recurrence with liver involvement and one ocular melanoma with pancreatic head and right liver lobe metastasis. All patients were submitted to pancreatoduodenectomy with a liver resection as follows: eight right trisectionectomies, five right lobectomies, four left lobectomies, 18 central lobectomies (IVb, V and VIII). The overall mortality was 34.2% (12/35) and the overall morbidity rate was 97.4%.

**Conclusion:**

Very high mortality is seen when major liver resection is performed with pancreatoduodenectomy, including right lobectomy and trisectionectomy. Liver failure in combination with a pancreatic leak is invariably lethal. Efforts to ensure a remnant liver over 40-50% of the total liver volume are the key to obtain patient survival.

## Abstract (pt)

**Racional:**

Hepatoduodenopancreatectomia é uma das operações abdominais mais complexas indicadas principalmente no carcinoma biliar avançado.

**Objetivo:**

Apresentar experiência de 10 anos executando esta operação em tumores malignos avançados.

**Métodos:**

Trata-se de estudo descritivo e retrospectivo. De 2004 a 2014, 35 hepatopancreatoduodenectomias foram realizadas em três diferentes instituições. A indicação mais comum foi carcinoma biliar avançado em 24 pacientes (68,5%).

**Resultados:**

Dezoito tinham câncer de vesícula biliar, oito tumores de Klatskin, cinco tumores neuroendócrinos com metástase hepática, uma metástase colorretal invadindo a cabeça do pâncreas, uma neoplasia mucinosa papilar intraductal com metástase hepática, uma recorrência do câncer gástrico com comprometimento hepático e um melanoma ocular com metástase na cabeça do pâncreas e no lobo direito do fígado. Todos os pacientes foram submetidos a duodenopancreatectomia com ressecção hepática da seguinte forma: oito triseccionectomias direitas, cinco lobectomias direitas, quatro lobectomias esquerdas, e 18 lobectomias centrais (IVb, V e VIII). A mortalidade global foi de 34,2% (12/35) e a taxa de morbidade global foi de 97,4%.

**Conclusão:**

Alta mortalidade é vista quando grande ressecção hepática é realizada junto à duodenopancreatectomia, incluindo lobectomia e triseccionectomia direita. Insuficiência hepática em combinação com deiscência do pâncreas é invariavelmente letal. Esforços para garantir fígado remanescente em 40-50% do volume total são a chave para obter sobrevida.

## INTRODUCTION

Hepatopancreatoduodenectomy (HPD) is a highly complex abdominal operation mainly used to treat advanced biliary carcinoma that includes gallbladder cancer and perihilar cholangiocarcinoma. In the western countries, it is in known as a "Japanese operation", and only few centers have developed interest in performing such major interventions in the treatment of very limited malignancies. Japanese specialized centers developed an enormous expertise in HPDs and delineated their role in treating advanced biliary carcinomas^5^^,^ 18 ^,^ 22. 

Advances in imaging technology over the last decade led to early diagnosis of gastrointestinal tumors. Patients, who do not share that fortune, typically present with jaundice and vascular encasement. The gallbladder cancers disseminate to local and distant lymph nodes by a richly drained submucosal layer of the biliary tree, especially at the body of the gallbladder, and by veins through the gallbladder fossa to intrahepatic portal branches of segment IV, V and VIII. The perihilar cholangiocarcinoma usually grow radially and locally with fewer distant disseminations routes such as those seen in gallbladder carcinomas^10^. 

Liver failure, pancreatic leakage and sepsis are the most catastrophic complications after HPDs. Prevention of these complications by systematic application of portal vein embolization, biliary drainage, bile replacement and preoperative nutritional optimization are of paramount importance^3^. 

The aim of this study was to present the results with this complex procedure, which represent the largest Western experience. 

## METHODS

This study was approved by the Ethics Committee of the Rio de Janeiro Adventist Hospital, Rio de Janeiro, RJ, Brazil

From March 2004 to September of 2014, 35 consecutive HPDs were performed in three institutions in Brazil. A retrospective analysis was based on chart revision. Most of the patients had advanced biliary cancers (68.5%) and all cases were performed by an experimented senior surgeon. 

### Procedures

All procedures include major liver resections (at least three segments) and pancreatoduodenectomy. The surgical approach always started with the pancreatoduodenectomy followed by the liver resection, mainly because vascular reconstructions were frequently applied (48.5%) and because the liver transection becomes easier without the pancreatoduodenal block. For biliary cancer an en-block specimen was always preferred. Indications of HPD are detailed in Table 1. 

**TABLE 1:** - Indications for hepatopancreatoduodenectomy

**Diagnosis**	**Indication**	**n**
Gallbladder cancer (n=18)	Peripancreatic lymph nodes metastasis	4
	Diffuse biliary infiltration	4
	Duodenal/pancreatic invasion	10
Klatskin tumor (n= 8)	Distal bile duct infiltration	3
	Direct pancreatic invasion	5
Neuroendocrine tumors (n=5	Duodenal invasion from recurred cancer	1
Colorectal metastasis (n=1) Metastatic intraductal papillary	Liver metastasis and pancreatic tumor	4
	Recurred liver tumor with duodenal invasion	1
Mucinous neoplasm (n=1)	Direct liver infiltration	1
Metastatic melanoma (n=1	Metastatic ocular melanoma	1
Gastric cancer recurrence (n=1)	Tumor invasion to liver and pancreatic head	1

Were separated those patients who underwent pancreaticoduodenectomy with right hepatectomy (Group 1), right trisectionectomy (Group 2), left hepatectomy (Group 3) and central hepatectomy (Group 4). Reconstruction of the pancreatic stump was made by three different technics: 1) pancreatojejunostomy in seven cases; 2) pancreato-gastrostomy in 17 cases, and 3) pancreatic stump ligation in 11 cases. A standard Roux-en-Y reconstruction of the biliary tree was performed on all cases.

## RESULTS

The overall 30-day mortality was 34.2% (12/35), independent of initial hospitalization time. Mortality was especially high in those patients who underwent major liver resections (Group 1 and 2 - Table 2). The most common causes of death were: liver failure as defined by Broek^27^, followed by abdominal sepsis related to pancreatic leakage, pneumonia, and renal failure. Groups 3 and 4 had lower complications rates and liver failure was not present in any of these patients. However, pancreatic leak was still a significant morbidity in this group. 

**TABLE 2:** - Types of liver resections on hepatopancreatoduodenectomies

**Liver resections**	**n**	**Mortality (%)**
Right hepatectomy	(Group 1) 8	5 (62.5%)
Right trisectionectomy	5 (Group 2)	2 (40%)
Left hepatectomy	(Group 3) 4	0
Central hepatectomy	(Group 4) 18	5 (22.7%)

Twenty-three patients who survived the surgical intervention and were discharged from the hospital, 11 cases were lost follow-up over the 10 years of this study. Twelve are alive with up to date follow-up (Table 3). Seven (20%) out the original cohort are alive and free of recurrence at the time of the writing of this report. 

**TABLE 3:** - Patients follow-up

**Age/Sex**	**Group Diagnosis Recurrence**	**Year**	**Diagnosis**	**Recurrence**	**Alive**	**Cause of death**	**Survival**
66M	1	2005	GBC	No	Yes	NA	9 years
70F	2	2006	Melanoma No	Yes	NA	8 years	
63M	2	2006	Hilar CC	Yes	No	Recurrence 39 months	
71F	3	2008	Hilar CC	No	No	Sepsis	15 months
69M	4	2008	GBC	No	Yes	NA	6 years
59M	1	2009	GBC	Yes	No	Recurrence 9 months	
61F	4	2010	GBC	Yes	No	Recurrence 27 months	
52M	4	2010	GBC	Yes	No	Recurrence 22 months	
56F	4	2012	GBC	No	Yes	NA	2 years
53F	4	2013	NET	No	Yes	NA	1 year
55M	3	2014	Gastric CA No	Yes	NA	1 year	
49F	4	2014	GBC	No	Yes	NA	6 months

GBC=gallbladder cancer; NET=neuroendocrine tumor; Hilar CC=hilar cholangiocarcinoma

## DISCUSSION

HPD was introduced in Japan in the 70´s to treat advanced biliary carcinoma^13^^,^ 21 ^,^ 25. Japanese specialized surgeons have developed significant experience in the surgical management of patients with advanced hepatopancreaticoduodenal cancer^1^^,^ 14 ^,^ 17 ^,^ 19 ^,^ 23. In the western hemisphere, very few groups are interested in reproducing this experience. So far, only Hemming et al.^9^ published a consisted report with HPD in the western countries. Nonetheless, a growing number of surgeons are engaged in developing more expertise with it in this decade. In the southern hemisphere, our groups have the largest HPD experience outside Japan.

Ebata et al.^6 ^define it as a multi-visceral resection combining a major liver resection and a pancreatoduodenectomy, which obtains an en-block specimen that encircles the entire extra-hepatic biliary system. In this study, all types of major liver resections with pancreatoduodenectomy performed simultaneously was called HPD^7^. In 1990´s Japanese reports with it, including Nimura´s initial series with 24 cases and 25% mortality, mainly by liver failure and sepsis^20^. Tsukada et al.^26^ reported seven HPDs with 29% mortality rate. Miyagawa et al.^16^ in 1996 published 0% mortality in seven cases and routine preoperative portal vein embolization. Shirai et al.^24^ in 1997 reported 17 cases in stage IV gallbladder cancer and the 5-years survival was 29% and those with R0 resection the 5-years survival was 50%. The authors learned from this series that HPD is a very complex operation and the combination of right lobectomy or right trisectionectomy has a high mortality rate and such procedures requires a mandatory portal vein embolization to ensure a liver remnant large enough to avoid liver dysfunction. Likewise, preoperative biliary drainage and bile replacement are also important tools to guarantee better clinical status. . The so called "Nagoya´s protocol" appears to be instrumental in order to achieve lower postoperative mortality18. The benefits of preoperative biliary drainage and bile re-infusion improving mitochondrial function, promoting adequate liver regeneration and also improving intestinal mucosa immunity to protect against bacterial translocation, has been well documented^2^^,^ 11.

Ebata et al.^4 ^reported that hepatic failure rates decreased from 56% to 14% after routine preoperative portal vein embolization. In the 2000´s, because portal vein embolization has been widely used in the preoperative management, a lower incidence of liver failure was observed and many reports with zero mortality following HPD. Interestingly, many studies showed a worse survival in gallbladder cancer when compared to cholangiocarcinomas. All this cumulative knowledge has encouraged many eastern surgeons to perform HPD in cholangiocarcinomas with very good results^9^. Ebata et al.^12^ reported a 10-years survival of 32.1% with it for cholangiocarcinomas. Kaneoka et al.^12^ and Miwa et al.^13^also reported excellent 5-years survival for HPD in cholangiocarcinomas 52% and 64%, respectively. Wakai, et al.^28^ in 2008 published 28 cases with perioperative mortality of 21%.

We acknowledge the weaknesses of this report where 11 patients (31.4%) were lost to follow-up. This is mainly related to the public health system in a very large country where patients need to travel long distances to get to specialized centers and many of those patients returned home were advanced medical care is scarce. Only 12 cases (34.2%) had long term follow-up in this series^8^.

During 10-years period, many improvements and experience were collected in the management of biliary carcinoma by the partners of this study. Using the Nagoya´s team protocol refined our approach to HPD. All the complications observed in this period were the same observed by Japanese surgeons in the 90´s. The combination of right trisectionectomy right lobectomy with pancreatoduodenectomy is feasible but mandatory portal vein embolization is required to obtain good postoperative survival because our results suggest that mortality over 40% is seen in those patients who underwent HPD (Group 1 and 2). So far, long term survival (9-years) was observed only in one patient who underwent right trisectionectomy plus pancreatoduodenectomy and portal vein reconstruction for gallbladder cancer and two patients with complex central hepatectomies plus pancreatoduodenectomy are also free of tumors recurrence in this series. Another four patients are alive and free of disease but the follow-up interval is short.

## CONCLUSION

HPD is a complex abdominal operation with a high mortality rate and a long learning curve. We emphasize that this operation should be limited to specialized centers with experience in liver transplantation background and advanced hepatobiliopancreatic surgery. Aggressive preoperative management with biliary drainage and portal vein embolization are crucial maneuvers to obtain satisfactory results. The HPDs for gallbladder cancer is feasible but requires a careful patient selection.
